# Peri-Prostatic Adipocyte-Released TGFβ Enhances Prostate Cancer Cell Motility by Upregulation of Connective Tissue Growth Factor

**DOI:** 10.3390/biomedicines9111692

**Published:** 2021-11-15

**Authors:** Evelina La Civita, Antonietta Liotti, Michele Cennamo, Felice Crocetto, Matteo Ferro, Pasquale Liguoro, Amelia Cimmino, Ciro Imbimbo, Francesco Beguinot, Pietro Formisano, Daniela Terracciano

**Affiliations:** 1 Department of Translational Medical Sciences, University of Naples “Federico II”, 80131 Naples, Italy; eva.lacivita@gmail.com (E.L.C.); antonietta.liotti@unina.it (A.L.); michelecennamo1@gmail.com (M.C.); pasquale.liguoro@uslsudest.toscana.it (P.L.); beguino@unina.it (F.B.); 2 Department of Neurosciences, Sciences of Reproduction and Odontostomatology, University of Naples “Federico II”, 80131 Naples, Italy; felice.crocetto@unina.it (F.C.); ciro.imbimbo@unina.it (C.I.); 3 Division of Urology, European Institute of Oncology, 20141 Milan, Italy; matteo.ferro@ieo.it; 4 Institute of Genetics and Biophysic, National Research Council, 80131 Naples, Italy; amelia.cimmino@igb.cnr.it

**Keywords:** adipocytes, prostate cancer, TGFβ1, peri-prostatic adipose tissue, cell migration

## Abstract

Periprostatic adipose tissue (PPAT) has emerged as a key player in the prostate cancer (PCa) microenvironment. In this study, we evaluated the ability of PPAT to promote PCa cell migration, as well as the molecular mechanisms involved. Methods: We collected conditioned mediums from in vitro differentiated adipocytes isolated from PPAT taken from PCa patients during radical prostatectomy. Migration was studied by scratch assay. Results: Culture with CM of human PPAT (AdipoCM) promotes migration in two different human androgen-independent (AI) PCa cell lines (DU145 and PC3) and upregulated the expression of CTGF. SB431542, a well-known TGFβ receptor inhibitor, counteracts the increased migration observed in presence of AdipoCM and decreased CTGF expression, suggesting that a paracrine secretion of TGFβ by PPAT affects motility of PCa cells. Conclusions: Collectively, our study showed that factors secreted by PPAT enhanced migration through CTGF upregulation in AI PCa cell lines. These findings reveal the potential of novel therapeutic strategies targeting adipocyte-released factors and TGFβ/CTGF axis to fight advanced PCa dissemination.

## 1. Introduction

Prostate cancer (PCa) is the most common tumor in male patients in Western countries [1]. Epidemiological data suggest a positive association between body mass index and advanced prostate cancer [2]. Excessive visceral adiposity corresponded to major probabilities of higher grade diagnosed PCa and poor clinical outcomes [3,4,5,6,7]. There is evidence that crosstalk with adipose tissue (AT) could affect PCa progression [8,9,10,11]. The prostate is encircled by periprostatic adipose tissue (PPAT). This fat layer is contiguous to the gland capsule [12], making it plausible that PPAT affects the prostate cancer malignant phenotype [13,14]. Accordingly, we recently demonstrated that a paracrine secretion of IGF-1 by PPAT reduced the docetaxel response of androgen-independent (AI) PCa cell lines [15]. Ribeiro et al. [14] showed that PPAT collected from obese patients was able to enhance migration of androgen-dependent (AD) and castration-resistant PCa cell lines.

Laurent et al. showed that PPAT stimulated extravasation of PCa cells by CCL7 release and extracapsular extension is a well-known predictor of PCa aggressiveness [16]. Accordingly, Sasaki et al. [17] demonstrated that the pre-therapy ratio between periprostatic and subcutaneous fat thickness can be useful as predictor of survival in men with advanced PCa treated with androgen deprivation therapy (ADT).

Thus, the molecular crosstalk between PPAT and PCa cells plays a crucial role in the prostate tumor microenvironment (TME) and might be the basis for more aggressive disease behavior.

In the present study we investigated the effects of adipocyte-released factors on PCa cell migration.

Clarifying the tumor-promoting factors secreted by PPAT and the underlying mechanisms of migration enhancement in PCa cells may allow the identification of prognostic biomarkers and therapeutic targets in patients with PCa.

## 2. Materials and Methods

### 2.1. Materials

Media, sera and antibiotics for cell culture were obtained from GIBCO (Thermo Fisher Scientific, Waltham, MA, USA). Antibodies against CTGF and glyceraldehyde-3-phosphate dehydrogenase (GAPDH) were obtained from Santa Cruz Biotechnology (Santa Cruz, CA, USA). Antibody against pSMAD2/3 (Ser 423/425) were obtained from Cell signaling Technology (Cell Signaling Technology, Danvers, MA, USA). Sodium dodecyl sulfate–polyacrylamide gel electrophoresis (SDS-PAGE) reagents were obtained from Bio-Rad (Hercules, CA, USA). All other chemicals were from Sigma-Aldrich (St Louis, MO, USA). Recombinant human TGFβ1 was purchased from R&D (R&D Systems Inc., Minneapolis, MN, USA), and SB431542 was purchased from MCE (MedChem Express, Monmouth Junction, NJ, USA).

### 2.2. Cell Cultures

LnCaP, DU145 and PC3 human prostate cancer cells were cultured in RPMI and DMEM, respectively, supplemented with 10% fetal bovine serum (FBS) and 2 mmol/L glutamine, 100 IU/mL penicillin and 100 IU/mL streptomycin. Cultures were maintained in a humidified atmosphere of 95% air and 5% CO_2_ at 37 °C. Human periprostatic adipose tissue (PPAT) samples were obtained from 14 men who had undergone radical prostatectomy at the Division of Urology of the University Federico II (Naples, Italy) from September 2020 to January 2021. All men were free from metabolic or endocrine diseases. Informed written consent was obtained from every study participant before the surgical procedure. This procedure was approved by the ethical committee of the University of Naples “Federico II” (protocol number 118/20). Periprostatic adipose-derived Mesenchymal Stem Cells (Ad-MSCs) were isolated from the Stromal Vascular Fraction and differentiated in mature adipocytes as previously described [18].

### 2.3. Conditioned Media System

Mature adipocytes were washed two times with sterile phosphate-buffered saline (PBS) and incubated with serum-free media supplemented with 0.25% albumin bovine serum (BSA). After 24 h, adipocyte-conditioned media (PPAT AdipoCM) were collected, centrifuged to remove cellular debris, and placed onto recipient cells.

### 2.4. Scratch Assays

LnCaP, DU145 and PC3 prostate cancer cell lines were seeded (5 × 10^5^ per well) in 12-well plates and allowed to adhere for 24 h. Confluent monolayer cells were scratched by a 200 μL pipette tip, washed three times with PBS to clear cell debris and suspension cells and a fresh medium was added. Cells were treated with the indicated stimuli and the cells were allowed to close the wound for 48 h. Photographs were taken at 0 and 48 h at the same position of the wound and the distance between the edges was measured.

### 2.5. Cell Transfection

DU145 and PC3 were transfected with Dicer-substrate RNAs (DsiRNAs, IDT Coralville, Coralville, IA, USA) by using Lipofectamine 3000 (Life Technologies, Carlsbad, CA, USA), in DMEM without antibiotics and serum, according to manufacturer’s instructions. After 6 h, the cells were feed with DMEM 10% FBS.

### 2.6. Real-Time RT-PCR Analysis

Total cellular RNA was isolated from DU145 and PC3 using QIazol reagent (QIAGEN Sciences, Hilden, Germany), according to manufacturer instructions. 1 μg of cell RNA was reverse-transcribed using Superscript III Reverse Transcriptase (Life Technologies, Carlsbad, CA, USA). PCR reactions were analyzed using IQTM SYBR Green Supermix (Bio-Rad, Hercules, CA, USA). Reactions were performed using Platinum SYBR Green qPCR Super-UDG using an iCycler IQ multicolor Real Time PCR Detection System (Biorad, Hercules, CA, USA). All reactions were performed in triplicate and PPIA was used as an internal standard. Primer sequences: human CTGF F: 5′ GGGAAATGCTGCGAGGAGT 3′, R: 5′ GATAGGCTTGGAGATTTTGG 3′; human PPIA F: 5′ TACGGGTCCTGGCATCTTGT 3′, R: 5′ GGTGATCTTCTTGCTGGTC 3′.

### 2.7. Western Blot

For Western blot assays, cells were washed with ice-cold phosphate-buffered saline (PBS) and harvested in a Laemmli buffer (with β-mercaptoethanol) containing a mixture of phosphatase inhibitors (0.5 mM sodium vanadate, 2 mM sodium pyrophosphate, 5 mM β-glycerolphosphate, and 50 mM sodium fluoride) and the protease inhibitor phenylmethylsulfonyl fluoride (Sigma–Aldrich). Western blots were carried out as previously reported [15].

### 2.8. TGFβ1 Elisa

AdipoCM levels of human TGFβ1 were evaluated using ELISA assay (Invitrogen, Carlsbad, CA, USA) according to manufacturers’ instructions.

### 2.9. Statistical Analyses

Statistical analyses were carried out using the GraphPad Prism software (version 9.0, C San Diego, CA, USA). Student’s t-test was used to compare the means of two groups, while a one-way ANOVA test was used to compare the means of more than two groups. All data are presented as the mean ± SD from at least three independent experiments. A two-sided *p*-value of less than 0.05 was considered statistically significant.

## 3. Results

### 3.1. PPAT Adipocyte-Conditioned Media-Induced Migration of Androgen-Independent PCa Cell Lines

PPAT samples were obtained from 14 men who underwent radical prostatectomy: 3 adenomas; 3 low-grade PCa [Gs ≤ 7(3 + 4)]; and 7 high-grade PCa [Gs ≥ 7(4 + 3)].

**Figure 1:**
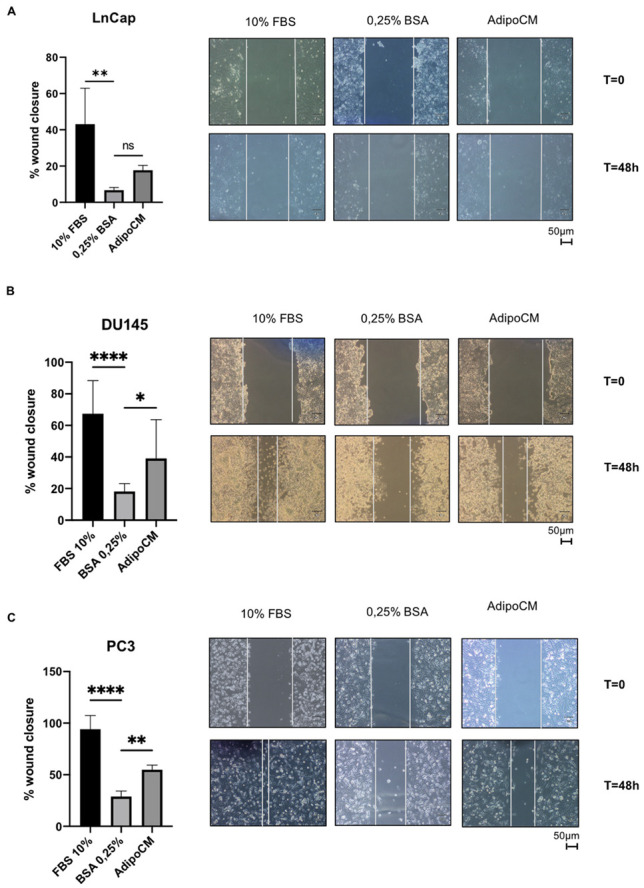
Adipocyte-conditioned media-induced PCa cell lines migration. LnCaP (**A**), DU145; (**B**) and PC3; (**C**) cells were seeded (5 × 10^5^ cells/well) in 12-well plates and allowed to form a confluent cell monolayer. Cell layers were wounded with a micropipette tip and then incubated with medium containing 10% FBS, 0.25% BSA or conditioned medium obtained from adipocytes. The images were acquired at 0 and 48 h using a camera connected to the microscope. Cell migration toward the wounded area was observed, photographed and measured (magnification 10×). Graphs show the percentage of wound healing rate. * Indicates a *p*-value < 0.05, ** indicates a *p*-value < 0.01 and **** a *p*-value ≤ 0.0001.

To investigate whether PPAT AdipoCM can influence cell motility in Pca cells, a series of wound-healing scratch assays were conducted on three different cell lines (LnCaP, DU145 and PC3). PCa cells were serum-starved and then incubated with AdipoCM from PPAT. As shown in Figure 1, AdipoCM incubation significantly enhanced migration in DU145 and PC3 androgen-independent cell lines, but not in hormone sensitive cell line LnCaP.

### 3.2. PPAT Mature Adipocyte-Released TGFβ1 Increased Cell Motility

**Figure 2:**
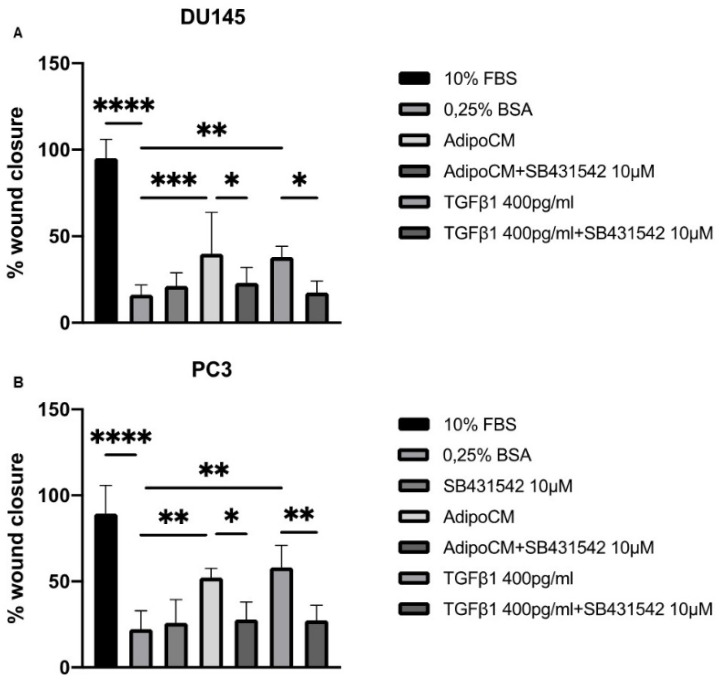
TGFβ1 receptor inhibitor counteracted the effect of AdipoCM on PCa cell lines migration. (**A**,**B**) DU145 and PC3 cells were seeded (5 x10^5^ cells/well) in 12-well plates and allowed to form a confluent cell monolayer. Cell layers were wounded with a micropipette tip and then incubated with medium containing 10% FBS, 0.25% BSA or conditioned medium obtained from adipocytes, human recombinant TGFβ1 (400 pg/mL) and SB431542 (10µM) alone or in combination. The images were acquired at 0 and 48 h using a camera connected to the microscope. Cell migration toward the wounded area was observed, photographed and measured (magnification 10×). Graphs show the percentage of wound healing rate. * Indicates a *p*-value <0.05, ** indicates a *p*-value < 0.01, *** indicates a *p*-value < 0.001 and **** a *p*-value ≤ 0.0001.

We speculated that TGFβ1 present in AdipoCM from PPAT could be the driver of increased migration capacity in DU145 and PC3 cell lines. To test our hypothesis, we performed scratch assays using SB431542, a potent and selective TGFβ1 receptor inhibitor (Figure 2). Interestingly, TGFβ1 receptor inhibitor reduced AdipoCM capacity to induce PCa cell migration (Figure 2A,B). Then, we measured the concentration of TGFβ1 in AdipoCM. As shown in Table 1, we found that mature adipocytes secreted TGFβ1 at a medium concentration of 336,4 ± 108,9 pg/mL. Accordingly, we stimulated PCa cell lines with human recombinant TGFβ1 at a concentration of 400 pg/mL, finding an effect comparable to AdipoCM. Our results suggest that TGFβ1 was the mediator of the motility-promoting effect on the PCa cells of PPAT adipoCM.

### 3.3. AdipoCM-Increased CTGF Expression

**Figure 3:**
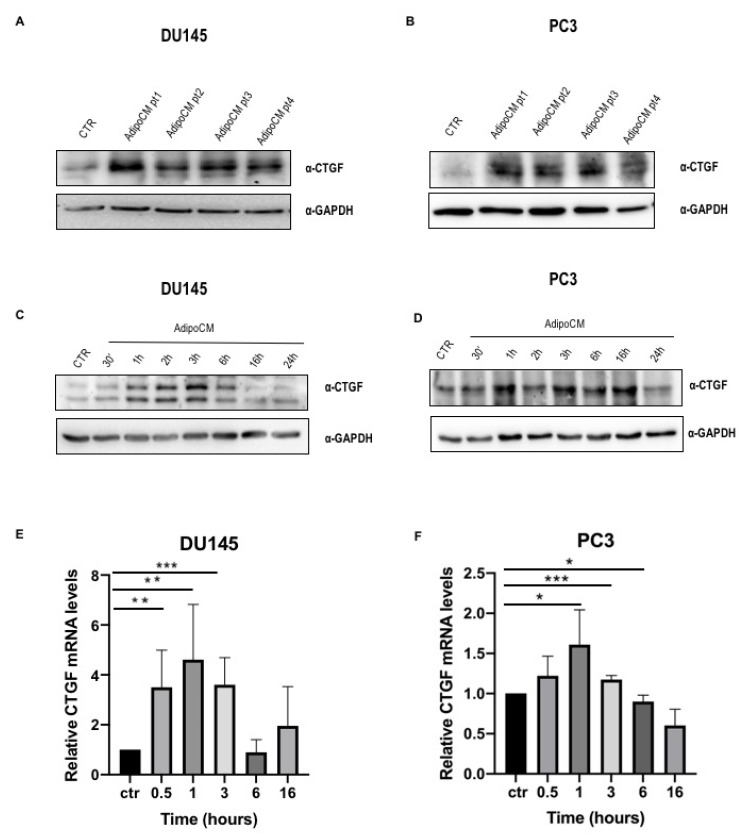
CTGF protein expression was upregulated by PPAT AdipoCM incubation. (**A**,**B**) DU145 and PC3 cells were seeded (2 × 10^5^ cells/well) in 12-well plates and incubated with serum-free media or AdipoCM from five different PCa patients. Cells were solubilized, and protein samples analyzed by Western blot with CTGF antibody. GAPDH antibody was used for normalization. Blot results were revealed by ECL and the autoradiograph is representative of three independent experiments. (**C**,**D**) DU145 and PC3 cells were incubated with PPAT AdipoCM at different time points, as indicated. Protein expression of CTGF was analyzed by Western blot using GAPDH antibody for normalization. (**E**,**F**) mRNA abundance of CTGF was measured by real-time RT–PCR analysis of total RNA using PPIA as internal standard with CTGF antibody. GAPDH antibody was used for normalization. * Indicates a *p*-value < 0.05, ** indicates a *p*-value < 0.01 and *** indicates a *p*-value < 0.001.

Thereafter, we investigated the downstream signal transductors of adipocyte-released TGFβ1. We began to consider AdipoCM’s ability to increase the expression of the connective tissue growth factor (CTGF). To investigate this, we incubated DU145 and PC3 cells with AdipoCM of four different patients, finding an increased CTGF protein expression (Figure 3). In addition, time course experiments showed that CTGF protein and mRNA expression was dynamically changed by AdipoCM incubation at different time points (Figure 3C,D), revealing a trend suitable for its involvement in migration.

### 3.4. CTGF Was the Intracellular Transductor of Adipocyte-Released TGFβ1

**Figure 4:**
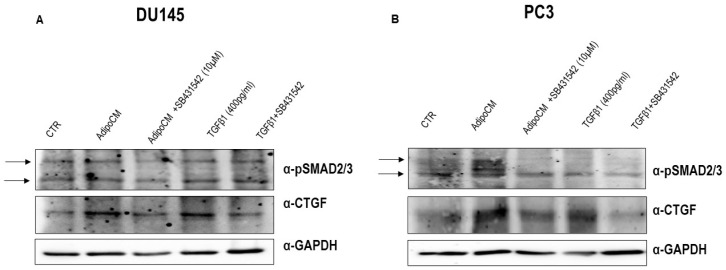
CTGF protein expression is reduced by TGFβ1 receptor inhibitor. (**A**,**B**) PCa cell lines were seeded (2 × 10^5^ cells/well) in multi-well plates and incubated with a medium containing 0.25% BSA or AdipoCM, human recombinant TGFβ1 (400 pg/mL) and SB431542 (10µM) alone or in combination. Cells were solubilized and protein samples analyzed by Western blot with CTGF and pSMAD2/3 antibodies. GAPDH antibody was used for normalization. Blot results were revealed by ECL and autoradiograph.

To test the hypothesis that CTGF could be the intracellular driver of adipocyte-released TGFβ1′s effect on PCa migration, we evaluated its protein expression after SB431542 pretreatment, in DU145 and PC3 cells. As shown in Figure 4, the incubation with SB431542 reduced the increase of CTGF induced by AdipoCM as well as by human recombinant TGFβ1 400 pg/mL.

The activation of TGFβ1 signaling was analyzed. Western blot analysis showed that AdipoCM incubation as well as human recombinant TGFβ1 increased phosphorylation of SMAD2/3. Accordingly, SMAD2/3 activation was decreased by SB431542 pretreatment (Figure 4A,B).

**Figure 5:**
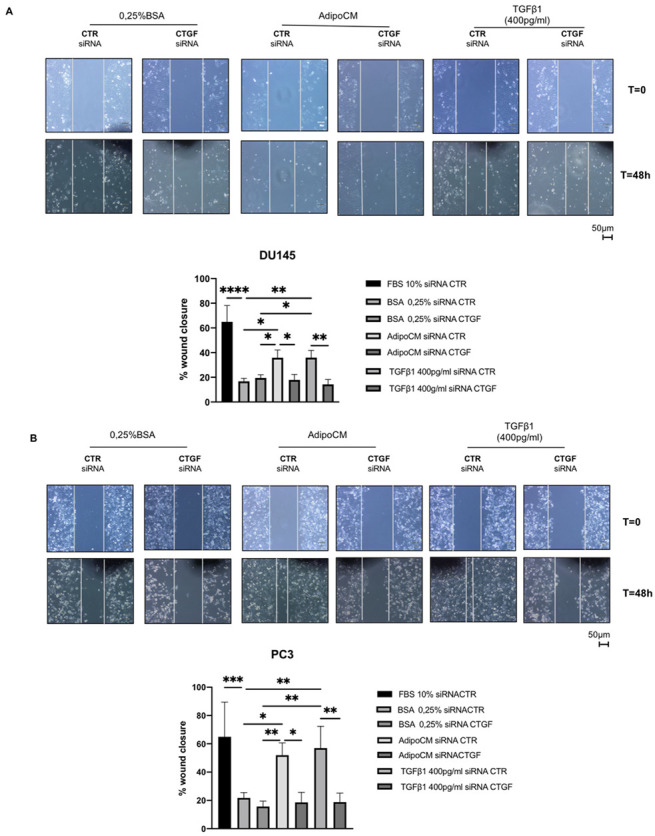
CTGF silencing counteracted the effect of AdipoCM on PCa cell migration. DU145 (**A**) and PC3 (**B**) cells were transfected with three different siRNAs recognizing CTGF (40 nM for DU145 and 10 nM for PC3; siRNA CTGF) or with a control siRNA (40–10 nM; CTR siRNA). After 6 h, the cells were fed with a complete medium. Then, the cells were allowed to form a confluent monolayer. Cell layers were wounded with a micropipette tip and then incubated with medium containing 0.25% BSA, conditioned medium obtained from adipocytes and TGFβ1 400 pg/mL. The images were acquired at 0 and 48 h using a camera connected to the microscope. Cell migration toward the wounded area was observed, photographed and measured (magnification 10×). Graphs show the percentage of wound healing rate. * Indicates a *p*-value <0.05, ** indicates a *p*-value < 0.01, *** indicates a *p*-value < 0.001 and **** a *p*-value ≤ 0.0001.

To further verify our hypothesis, we performed scratch assays silencing CTGF. Firstly, we checked CTGF knockdown by Western blot (Figure S1), confirming that CTGF siRNA blocked its protein expression in both PCa cell lines. Secondly, we demonstrated that CTGF silencing significantly blocked the wound closure induced by AdipoCM in both PCa cell lines (Figure 5).

## 4. Discussion

**Figure 6:**
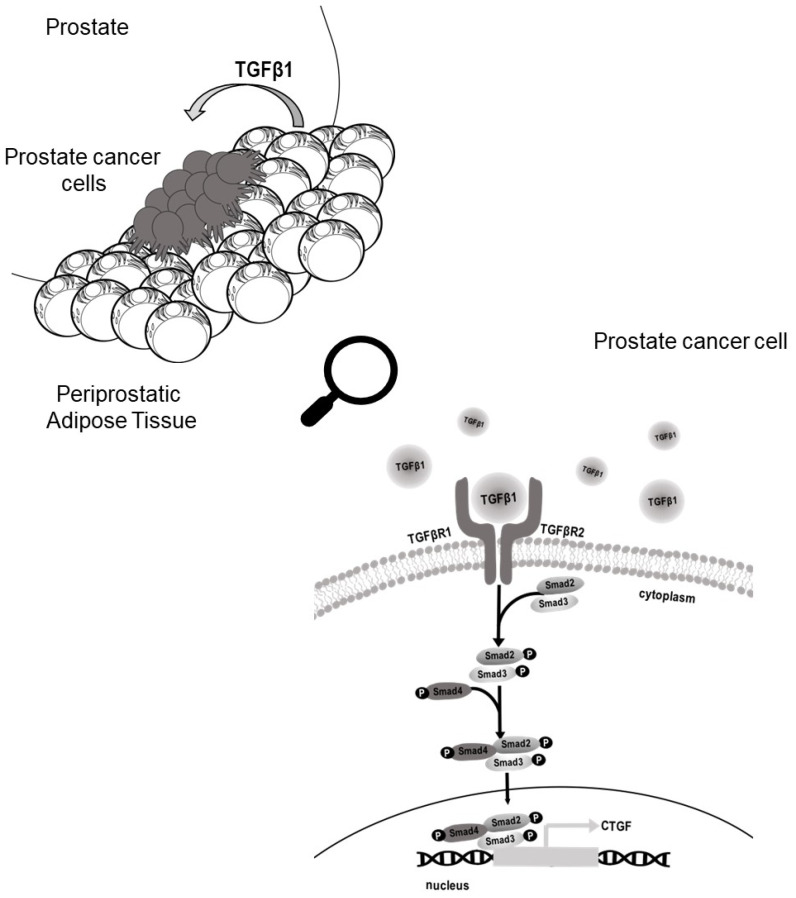
Schematic representation of the proposed role of PPAT in promoting migration in PCa cells. Periprostatic mature adipocytes released TGFβ1 upregulated CTGF expression in PCa cells favouring migration. CTGF, connective tissue growth factor; PCa, prostate cancer; PPAT, periprostatic adipose tissue; TGFβ, tissue growth factor β.

The molecular mechanism linking PCa and AT is not completely clarified [19]. As there is a rising prevalence of obesity, understanding the underlying mechanisms of this biological connection is urgently needed. At present, obese and lean subjects receive the same treatment. However, obese patients had a poorer clinical outcome [20]. Thus, personalized therapeutic strategies based on patients’ BMI or AT-associated measures may ameliorate their survival. To address this issue, it could be informative to investigate the effect of PPAT-released factors on the PCa cell aggressive phenotype. We previously demonstrated that adipose tissue-released IGF-1 contributed to docetaxel resistance of PCa cells [15]. In addition, the ability of adipocyte secretome to promote metastatic expansion has been clearly demonstrated in breast cancer [21]. There is growing evidence that adipocytes promote the malignant behavior of cancer cells [22]. Here, we show that AdipoCM from PPAT promotes migration in AI PCa cells through TGF-β upregulation of CTGF (Figure 6).

This finding was consistent with previous research, since the role of TGF-β in PCa is well-known and dual: it functions as anti-proliferative stimulus in the early stages, and then becomes a pro-metastatic factor in the advanced stage [23,24,25,26].

For this feature, TGF-β was defined as the molecular “Jekyll and Hyde” of cancer [27] and, despite TGFβ signalling complexity in the tumor microenvironment, it can be targeted for therapeutic intervention.

Several studies showed that TGF-β promoted migration of PCa cells, suggesting a strong association between TGF-β signalling and PCa progression [28,29,30,31]. Ribeiro et al. [14] previously demonstrated that factors produced by AT explants significantly increased the migration of both hormone-refractory PC-3 and hormone-sensitive LNCaP cells. Contrarily to Ribeiro et al. [14], we used isolated adipocytes rather than whole ATs and we did not find effects on LNCaP cell migration. Our model allowed us to isolate the role of factors secreted from adipocytes, whereas the ex vivo method using explants revealed the effects of all the cellular components included in AT, including mature adipocytes, pre-adipocytes, fibroblasts and immune cells. It seems that adipocyte-released factors enhanced androgen-independent but not androgen-dependent motility. Conversely, whole adipose tissue secretion promoted the migration of both cells. It is plausible that the effect on androgen-sensitive cells is mediated by a different driver from TGF-β and released by cellular components other than adipocytes. The lack of a migration-promoting effect of Adipo-CM on LNCaP was in agreement with the anti-oncogenic role played by TGFβ signalling in epithelial, but not in mesenchymal, cells [27]. Accordingly, higher expression of TGF-β was reported in tumor tissues with higher Gleason score [32]. It is well accepted that TGF-β affects the expression of several stromal derived factors involved in tumor progression, including CTGF through Smads [33,34]. Several authors reported that CTGF interacts with various integrins (α2β1, α5β1, αvβ6, αvβ1) in normal cells [35,36,37]. In breast cancer cells, CTGF increased cell viability and migration via an integrin-αvβ3 pathway [38,39]. Interestingly, upregulation of integrin-αvβ3 has been reported in bone metastatic cancer [40]. In addition, the role of CTGF in promoting the ability of breast and prostate cancer cells to establish bone metastasis has been recently demonstrated [41]. Moreover, protein interacting with PRKCA 1 (PICK1), a negative regulator of the TGF-β pathway, can repress prostate cancer metastasis to bone [42]. In human PCa tissues, CTGF is upregulated in the advanced stage [43], highlighting the clinical relevance of our findings.

Here, we show, for the first time, that PCa cell migration was enhanced by CTGF-increased expression induced by adipocyte-released TGF-β, providing insights to the hypothesis that the TGF-β/CTGF axis is a relevant mediator of the crosstalk between PCa and AT.

TGF-β in PPAT may act in a paracrine manner, regulating function of the neighbor cells, including PCa cells. Accordingly, we showed that inhibition of the TGF-β receptor by SB431542 decreased the effect of adipocyte-released factors on migration, thereby indicating that TGF-β is a pivotal factor in the adipocyte regulation of PCa cell motility.

Our results are limited to an in vitro interaction model between PCa cells and adipocytes. Further studies are needed to assess the relevance of TGF-β released by PPAT in the regulation of migration in PCa. However, our study strengthens the hypothesis that distinct AT (e.g., PPAT) may promote cancer dissemination [44] more than BMI. Accordingly, there is growing evidence that BMI did not mirror the role played by each type of AT [45]. Our findings reinforce the utility of the model measuring PPAT thickness rather than calculating the BMI to obtain clinically relevant information on PCa prognosis [17,44]. It could be valuable to combine PPAT-associated measures with PCa risk calculator to further improve PCa prognosis evaluation and to choose an individualized therapeutic strategy. Our results provided insight into molecular basis on the relationship between PPAT and PCa progression and envisioned the potential use of CTGF as a prognostic biomarker and the TGF-β receptor as potential therapeutic target in patients with metastatic PCa. CTGF could be a reliable biomarker that will enable clinicians to choose the best therapeutic strategy for each individual PCa patient. Notably, there are several anti-cancer pharmacological agents that target the TGF-β pathway that have already been tested in clinical trials for advanced PCa [46].

## 5. Conclusions

Increased migratory capacity is undoubtedly a hallmark of an unfavourable prognosis. Our results demonstrated for the first time in PCa that tumor-surrounding adipocytes promote migration through a CTGF-dependent mechanism, highlighting the relevant role played by PPAT on PCa clinical outcome and offering new chances to develop personalised treatment for patients with advanced PCa.

## Figures and Tables

**Table 1:** Clinicopathological characteristics of patients.

Low Grade Patients				
Patient Code	Age (Years)	BMI (kg/m^2^)	Grading (Gleason Score)	Adipo-CM TGFβ1 (pg/mL)
1	67	25	6 (3 + 3)	262.9
2	66	31	7 (3 + 4)	303.4
3	65	32	7 (3 + 4)	250.8
**High grade patients**				
**Patient Code**	**Age (years)**	**BMI (kg/m^2^)**	**Grading** **(Gleason Score)**	**Adipo-CM TGFβ1 (pg/mL)**
4	75	25	7 (4 + 3)	406.9
5	77	23	8 (4 + 4)	396.9
6	72	23	8 (4 + 4)	259.0
7	77	26	8 (4 + 4)	376.2
8	58	26	8 (4 + 4)	501.9
9	69	24	8 (4 + 4)	382.6
10	73	29	9 (4 + 5)	465.7
11	74	28	9 (4 + 5)	478.0
**BH patients (controls)**				
**Patient code**	**Age (years)**	**BMI (kg/m^2^)**		**Adipo-CM TGFβ1 (pg/mL)**
12	71	27	-	158.8
13	66	37	-	284.1
14	70	25	-	183.1

## Data Availability

The datasets used and/or analyzed during the current study are available from the corresponding author on reasonable request.

## Supplementary Materials

The following are available online at https://www.mdpi.com/article/10.3390/biomedicines9111692/s1, Figure S1: Western blot analysis of CTGF silencing in DU145 and PC3 cells lines.

## Abbreviations

PPAT: Peri-Prostatic Adipose Tissue; AdipoCM: Conditioned Medium of human PPAT; PCa: Prostate Cancer; CTGF: Connective-Tissue Growth Factor; AT: Adipose Tissue; BMI: Body Mass Index; TGF-β: Transforming Growth Factor-β; TME: Tumor Microenvironment; DMEM: Dulbecco’s Modified Eagle Medium; FBS: Fetal Bovine Serum; AdMSCs: Adipocytes Mesenchymal Stem Cells; PBS: Phosphate-Buffered Saline; BSA: Bovine Serum Albumin; GAPDH: Glyceraldehyde-3-Phosphate Dehydrogenase; ELISA: Enzyme-Linked Immunosorbent Assays; TBS: Tris-Buffered Saline; mCRPC: Metastatic Castration-Resistant Prostate Cancer; EMT: Epithelial–Mesenchymal Transition; ADT: Androgen Deprivation Therapy; PPIA: Peptidylprolyl Isomerase A; SMAD2/3: Sma- And Mad-Related Proteins 2/3; AI: Androgen-Independent.
