# Supplementary material for: Peri-Prostatic Adipocyte-Released TGFβ Enhances Prostate Cancer Cell Motility by Upregulation of Connective Tissue Growth Factor
Source: Biomedicines. 2021 Nov 15;9(11):1692. doi: 10.3390/biomedicines9111692 (PMC8615771; doi:10.3390/biomedicines9111692)
Supplement: Supplementary file 1 [file biomedicines-09-01692-s001.zip › biomedicines-1461022-SI.pdf]

Western blot analysis of CTGF silencing in DU145 and PC3 cells lines

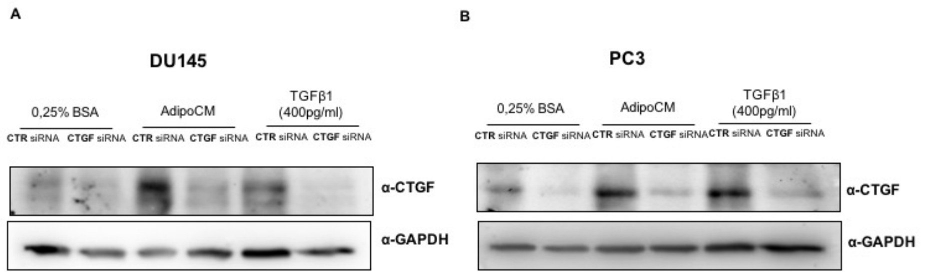

Figure S1. CTGF protein expression is downregulated by CTGF siRNA. (**A**, **B**) PCa cell lines were seeded (2×10^5 cells/well) in multi-well plates, transfected with three different siRNAs recognizing CTGF (40 nM for DU145 and 10 nM for PC3; siRNA CTGF) or with a control siRNA (40–10 nM; CTR siRNA). After transfection DU145 and PC3 were incubated with a medium containing 0.25% BSA or AdipoCM or human recombinant TGFβ1 (400 pg/mL). Cells were solubilized and protein samples analyzed by Western blot with CTGF antibody. GAPDH antibody was used for normalization. Blot results were revealed by ECL and autoradiograph.
